# Religio Medici, and Other Essays

**Published:** 1898-12

**Authors:** 


### Reviews of Books.

**Religio Medici, and other Essays.** By Sir THOMAS BROWNE. Edited by D. LLOYD ROBERTS, M.D. Pp. xxxix., 305. London: Smith, Elder and Co. 1898.

However laudable a project it may be to attempt to revive the popularity of Sir Thomas Browne's writings, we confess that we are not very sanguine of its success. Dr. Lloyd Roberts has been at much pains to put forth a correct and handy edition of some of his works, or "pieces" as the author would have called them, in which the marginal epitome and references, doubtless reprinted from a valuable original to which he refers, do much to assist the reader. In the biographical introduction, which is by no means the least interesting part of the book, we have all the little that is to be learned of the placid life of our author. Born in 1605, he lived through a most stirring period of English history; and yet in all his writings he makes scarcely a single allusion to passing events. He lived, as Dr. Roberts observes, oblivious "to the stupendous dramas enacted all around him." Sir Thomas Browne appears to have been a profound classical scholar and antiquarian rather than a man of science. In acknowledging some of the difficulties which present themselves to his understanding in the study of Holy Scripture, he triumphs in the victory he had obtained not by reason but by faith. "Active faith" is the keynote of the *Religio Medici.*

Of the other essays contained in this volume, although the *Hydriotaphia* or *Urn Burial* ranks highest in general estimation, we think the *Letter to a Friend* will be read with greatest interest by the members of Sir Thomas's profession. In it he speculates as to the hour of birth and of death, and season of the year at which death is most prevalent—"that mortal time," he calls it, "when the leaves of the fig-tree resemble a daw's claw." And he gives us a number of curious clinical and *post-mortem* observations, notably one of very extensive pleuritic adhesions, the patient having suffered from gout and died of stone, and "thus brake the rule of Cardan" that they that suffer from gout "are delivered thereby from the phthysis and stone in the bladder." In this letter our author appears as the kindly and observant physician rather than the pedantic scholar and philosopher. In a fanciful passage of it he speaks of the unlikelihood of man completing a cycle of years and dying on the anniversary of his birth, like a serpent with its tail in its mouth. And in *Urn Burial* he commends cremation as a preventative of the gruesome offence of body-snatching. Curiously enough both these fates befell Sir Thomas Browne; for he died on the 19th of October, 1682, the anniversary of his birth, and he was "knav'd out of his grave" and his skull placed in the museum of Norwich Hospital.

The manner in which these essays are here presented offers a direct incentive to perusal. On the same lines, an edition of the *Pseudodoxia Epidemica* would be acceptable.

